# Genetic Investigation of Bisphosphonate-Related Osteonecrosis of Jaw (BRONJ) via Whole Exome Sequencing and Bioinformatics

**DOI:** 10.1371/journal.pone.0118084

**Published:** 2015-02-10

**Authors:** Jee-Hwan Kim, Yong Jae Ko, Ji-young Kim, Yoonsoo Oh, Jihye Hwang, Sangjin Han, Sanguk Kim, Jae-Hoon Lee, Dong-Hoo Han

**Affiliations:** 1 Department of Prosthodontics, College of Dentistry, Yonsei University, Seoul, Korea; 2 Department of IT Convergence and Engineering, Pohang University of Science and Technology, Pohang, Korea; 3 Department of Life Sciences, Pohang University of Science and Technology, Pohang, Korea; 4 Department of Prosthodontics, College of Dentistry, Yonsei University, Seoul, Korea; Uppsala University, SWEDEN

## Abstract

Complications associated with the use of bisphosphonate (BP) have risen over the years due to an increase in the prescription of BP. BP-related osteonecrosis of jaw (BRONJ), one of the complications linked to the consumption of BP, greatly affects patients with minor dental trauma, incurring a long healing period. While BRONJ afflicts only a minority of patients prescribed with BP, BRONJ is a multigenic disease affected both by environmental and genetic factors having a distinctive phenotype. This study aims to discover genetic biomarkers associated with BRONJ via whole exome sequencing (WES) followed by statistical analysis. Sixteen individuals who had been prescribed with bisphosphonate medication and diagnosed as BRONJ were chosen and each individual’s saliva sample was collected for WES. 126 randomized subsamples from the GSK project representing 109 male and 17 female Koreans were used as a control data set. Fisher’s exact test was carried out to assess the significance of genetic variants in BRONJ patients. Gene set enrichment analysis (GSEA) (DAVID Bioinformatics Resource 6.7) was used to perform a cluster analysis of variants found from Fisher‘s exact test. The results from this study suggest that BRONJ-inducing factors are genetically associated and BRONJ occurs due to the malfunctioning of post-translational modification in osteoclast leading to the impairment of cell morphology and adhesion.

## Introduction

Bisphosphonates (BP) are a commonly prescribed medication to treat bone metastases, multiple myeloma, osteoporosis, and other bony diseases [1,2,3]. It is prescribed at 73 percent of physician visits for osteoporosis in the United States [4]. No significant side effects have been reported but patients prescribed with BP over a long period tend to experience complications during healing after minor trauma in dentistry such as tooth extraction, periodontal surgical operation, and tooth operations.

In 2003, it was first reported that bisphosphonate-related osteonecrosis of jaw (BRONJ) derives from the exposure and necrosis of alveolar bone, pain, infection, and abscess formation [5]; several other cases have since followed [6,7,8,9]. Many groups have recently published recommendations or guidelines on prevention, staging, and management strategies for BRONJ [10,11,12,13,14,15,16,17,18,19,20,21]. Nevertheless, much needs to be done concerning the incidence, pathogenesis, treatment, and prevention of BRONJ.

Patients who have received or been exposed to bisphosphonate and have not had craniofacial region radiation therapy can be diagnosed with BRONJ if they have exposed jaw bone that has not healed within 8 weeks after identification by a health care provider. The 8-wk duration is consistent with the time frame in which most trauma, extractions, and oral surgical procedures would have resulted in soft tissue closure and exposed bone would no longer be present [22].

The incidence of the disease seems to be relatively low in patients receiving oral bisphosphonates for osteoporosis or Paget’s disease and considerably higher in patients with malignancy receiving high doses of intravenous bisphosphonates. The mean incidence after intravenous application was 7% and the overall incidence of BRONJ after oral bisphosphonate application was 0.12% [23]. In a clinical investigation of BRONJ in patients with malignant tumors, the disease recurred at the same sites in 7 out of 20 patients (37%) and at different sites in 3 patients (16%) [24]. Not all patients receiving BP treatment experience BRONJ, a clinical study showing an estimated risk of between 0.8% and 12% [25]. These varying statistical values imply that BRONJ is a multifactorial disease involving several factors in combination.

BP is known to inhibit osteoclastic bone resorption via attraction to and localization in areas of the bone undergoing inflammation or resorption. Recently, substantial evidence has emerged supporting such actions of BPs. Nitrogen-containing BPs are subsequently phagocytized and internalized by osteoclasts, wherein they inhibit the mevalonate pathway during cholesterol synthesis [26]. Such obstruction causes impairment of small GTPases of the Ras family, which are known to be involved in cytoskeletal activity of bone-resorbing osteoclasts [26]. The internalized BPs triggers apoptosis of osteoclasts, inhibiting osteoclast-mediated bone resorption [27,28].

Several studies on BRONJ-linked environmental risk factors have examined issues such as the use of intravenous vs. oral BPs [29], concomitant use of chemotherapy [30], treatment with glucocorticoid [10] or thalidomide [31], length of exposure to BP treatment [32,33,34], the presence of comorbid conditions such as obesity [35,36], alcohol and/or tobacco abuse and pre-existing dental or periodontal disease. Among these, dental trauma such as tooth extraction is known to be the most common immediate precipitation risk factor [37].

Other predisposing factors for BRONJ are age, race, smoking, obesity, cancer diagnosis, and poor oral health, though these only account for a small percentage of the entire risk [32,35,38]. Since patients with BP medication undergo similar biological effects due to the intake of BP and considering that only a small number of BP users experience BRONJ, it can be hypothesized that genetic susceptibility is conferred by multiple genes regulating the metabolism of BP or skeletal homeostasis with small variations [39]. If so, BRONJ, like many other complex trait diseases, may be caused by a combination of environmental and genetic risk factors.

Previous genetic association studies found various genes such as vascular endothelia growth factor (*VEGF*), collagen Type 1 A 1 (*COLIAI*), cytochrome P450 subfamily 2 polypeptide 8 (*CYP2C8*), farnesyl disphosphate synthase gene, Matrix metalloproteinase-9 (*MMP9*), and peroxisome proliferator-activated receptor gamma (*PPARG*) [40,41,42,43,44,45,46,47,48,49] to be associated with risk of developing BRONJ. Until 2004, genetic research depended on advanced technologies and case control studies primarily identified only a small number of variants related to BRONJ.

Case-specific approaches have attempted to accommodate small case numbers. The first genome-wide association study (GWAS) reported the rs1934951 (*CYP2C8)* single nucleotide polymorphism (SNP) was associated with BRONJ in multiple myeloma (MM) [50]. However, two other studies reported that this SNP showed no correlation with jaw osteonecrosis in patients suffering from prostate cancer and neither research group could confirm a significant association between polymorphisms in the *CYP2C8* gene and the risk of developing osteonecrosis of the jaw in patients with MM receiving treatment with BP in an independent series [51,52]. So far, no single gene has been verified as a risk factor despite numerous GWAS studies. This is due to the limitation of GWAS in representing SNPs when only five thousand to one million bases out of three billion human base pairs are analyzed. Newly discovered genetic indicators revealed the limits of GWAS and gave rise to many discussions regarding missing heritability in GWAS. A relatively new method, next generation sequencing (NGS), accommodates these limitations.

In previous studies, a SNP array is commonly used to identify variants within a certain range. One of the limitations of SNP arrays is that the analysis is done using the preexisting reference SNP. The goal of this study is to find novel variants and to include them in our analysis of both rare and common variants relating to BRONJ. Therefore, Whole exome sequencing (WES), NGS technology, is more appropriate for the purpose of this study.

NGS technology shifted genetic research from investigating known candidate genes to revealing gene mutations and discovering candidate genes by comparing case and control. Because NGS targets the exome, mutations in non-synonymous variants, splice sites, and coding indels can be identified, particularly by focusing on non-synonymous mutations in which changes in amino acids affect protein function. However, WES alone may not provide pragmatic results in a multi-genic disease like BRONJ due to the extensive raw data, pointing out the need to integrate data management and computational screening. Incorporation of Gene Set Enrichment Analysis (GSEA) and a protein functional network study of WES data may define enriched functions related with genetic variants implicitly related to BRONJ. GSEA provides a novel way to functionally analyze a large number of variants in a high-throughput fashion by classifying them into gene groups based on their annotation term co-occurrence. The objective of this study was to discover genetic biomarkers associated with BRONJ via WES GSEA, as well as network analysis followed by statistical analysis and comparison with known genes.

## Material and Methods

### 1. Ethics Statement

All research involving human subjects or human data was approved by the Institutional Review Board of Yonsei University College of Dentistry (Yonsei IRB No. 2–2014–0018). All clinical investigation was performed in accordance with the Declaration of Helsinki. Written informed consent was obtained from all subjects prior to participation.

### 2. Patient Selection

Sixteen individuals between 55 and 90 with BRONJ were analyzed using massively parallel sequencing in this study (1 male, 15 female). Sixteen individuals had tooth extraction or implant surgery in the Implant Clinic of Yonsei University Dental Hospital from 2008 to 2013. These patients had a history of bisphosphonate medication with varying duration, presence of exposed bone in the maxillofacial region for more than eight weeks, and no history of radiation therapy to the jaws.

### 3. Control Data Set

126 randomized subsamples from the GSK project (Koreans; 109 male and 17 female) were used as the control data set. The subsamples from the reference population consisted of healthy Koreans regardless of gender and age originally recruited for a thyroid cancer study (GSK project) (S1 Table).

### 4. Sample Collection

The sixteen individuals participating in this study were asked to collect 2 mL of saliva in the tube of an Oragene DNA Self-Collection kit (DNA GenoTek, Ottawa, Ontraio, Cat. #OG-500). DNA-preserving solution was mixed with the saliva, which was sent to DNA Link Inc. (Seoul, South Korea) where collection of genomic DNA, extraction of DNA, and further analysis were completed.

### 5. Whole Exome Sequencing on HISEQ 2500 using SureSelect All Exon kit 50Mb

Whole Exome Sequencing was done following the protocol reported in a previous study [53].

### 6. Whole Exome Sequencing and variant analysis

Sequence QC was done through FastqQC 0.10.1, and then mapped to human reference genome sequence NCBI b37 using bwa v0.7.5a. BAM files were realigned with the Genome Analysis Toolkit 2.8–1 (GATK) IndelRealigner, and base quality scores were recalibrated using the GATK base quality recalibration tool. Variants were called with GATK’s UnifiedGenotyper tool. In order to filter potential errors, GATK Variant Quality Score Recalibration (VQSR) was conducted based on hapmap 3.3, NCBI Variation Database (dbSNP138), 1000 genome, and an Omni 2.5M SNP chip array. Then, the variants’ functional information was annotated using SnpEff v3.6h with the GRCh37.75 reference set. Variants found were then processed to find impact variants, i.e., moderate and high variants as well as variants with call rates over 90% (S2 Table). High impact variants are those variants that have a disruptive impact in protein and are likely to affect the function of the protein, whereas moderate impact variants are those that may or may not affect the protein. Genomic Evolutionary Rate Profiling (GERP) and Polymorphism Phenotyping v2 (polyPhen-2) scores were applied to the variants that matched the criteria and those that showed significance were subject to further statistical analysis. For samples showing no result, a GERP greater than 2, or Polyphen-2 results with D were filtered during this analysis.

### 7. Statistical analysis

Fisher’s exact test was carried out to assess the significance of the variants in BRONJ patients. Allelic, dominant, and recessive models were tested and variants with p-value < 0.05 were chosen for each model. In this study, a 0.05 cutoff indicates a broad view of association between the phenotype and genotype in the data. The results were examined for multiple testing problems. Bonferroni correction with a stricter cutoff of 3.75E-6 for statistical significance found significant variants. Initially 15 variant genes were obtained by using Bonferroni correction with a stricter cut-off (p-value< 3.75E-6). However, the initial gene set obtained was not sufficient for the function enrichment analysis. In this study, rather than analyzing the direct effect of disease genes, the relationship between genetic variants in a functional network were analyzed. Therefore, the initial gene set was expanded for further functional network analysis with a less strict cut-off (p-value <0.05). As a result, 201 variant genes were obtained for the analysis of functional association of pathogenesis of BRONJ. The significant SNPs were not emphasized with Bonferroni correction because the main focus of the analysis was to find functional significance rather than statistical significance with testing. A call rate of greater than 90% was used. Three different analysis models (dominant, recessive, and allelic) were used to compare genotype frequencies. In a dominant model, a group of homozygote of the major frequency allele (A) was compared with homozygote of minor allele (B) plus heterozygote (AA vs. AB+BB). In contrast to the dominant model, the recessive model compared a group of homozygote of major allele and heterozygote with a group of homozygote of minor allele (AA+AB vs. BB). Finally, the allelic model compared the number of major and minor alleles (A vs. B) in cases and controls.

### 8. Gene set enrichment analysis

Gene set enrichment analysis (DAVID Bioinformatics Resource 6.7) was used to perform a cluster analysis of variants found through Fisher’s exact test. GSEA was applied to investigate genetic variants in groups of genes sharing a common biological function, domain, or pathway. With the GSEA results, cluster enrichment analysis was performed to build a protein functional network. The following categories in DAVID were used. In the ‘‘Functional Categories” section, ‘‘COG ONTOLOGY” and ‘‘UP SEQ FEATURE;” in the ‘‘Gene Ontology” section: ‘‘GOTERM BP FAT”, ‘‘GOTERM CC FAT,” and ‘‘GOTERM MF FAT;” in the ‘‘Protein Domains” section, ‘‘INTERPRO,” “PIR SUPERFAMILY,” and ‘‘SMART;’ and finally, in the ‘‘Pathways” section, ‘‘KEGG PATHWAY” was used. If the cluster had more than one annotation term in “Initial Group membership” of Classification Stringency Options, “Final Group Membership” was adjusted to “2” in order to set the cluster.

### 9. Construction of a protein functional network

**Fig 1:**
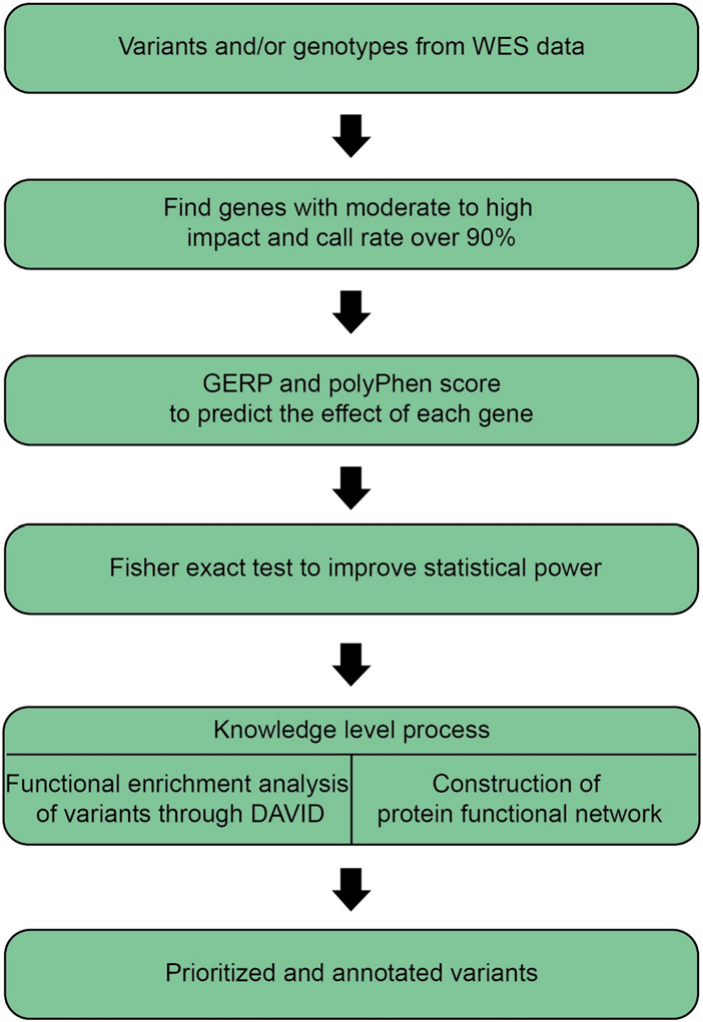
An outline of variant selection for data analysis.

A protein functional network was visualized using the top ten clusters of the highest enrichment scores. If different terms shared two or more genes, a link was made. Link thickness represents the number of genes shared among distinct terms and node size shows the number of genes in each term. Fig. 1 outlines the filtration and prioritization framework used for data analysis.

## Results

All individuals had a history of BP medication with varying duration. Information regarding all 16 individuals is provided in S3 Table. An average of 67,035,644 reads and 6,771 megabases were obtained from the sixteen individual’s WES results. An average of 4,138,925,783.75 total bases was aligned with a mean coverage depth of 80.36. All information regarding number of reads, sample coverage, and sequencing depth, as well as data quality, is summarized in S4 Table.

A total of 142 samples (16 case samples and 126 Korean GSK samples) were used for the initial variant call. The selection yielded 219,722 variants, which were then processed to find impact variants (moderate and high variants as well as variants with a call rate over 90%). 69,187 variants were found to match the criteria, 13,325 variants showing significance based on GERP and polyPhen scores. Fisher’s Exact test was used to improve statistical power and 201 variants were found to have a p-values < 0.05 (S5 Table). Variants chosen were selected for subsequent gene set enrichment analysis. All whole exome sequencing raw data was submitted to the SRA database (SRA, http://trace.ncbi.nlm.nih.gov/Traces/sra/, accession number SRP045344). Variants with affected number = 0 were included in the data analysis because the conserved sequences in patients data showed meaningful differences compared to the control set. The genes with “affected number = 0” have conserved sequences in our 16 patient dataset while in the 126 control dataset, the same genes showed multiple variations in the sequences [54,55].

DAVID (DAVID v6.7) was used to canvass BRONJ pathogenic genes for enriched functional-related gene groups. GSEA detected multiple gene sets related to cell adhesion, regulation of cell morphology, and post-translational modification. Protein functional network analysis was then performed to see how genes from different domains related with each other in a biological system and how interactions between genes might affect pathogenesis of BRONJ. To better understand the function of genetic variants from BRONJ patients, we searched for significantly enriched gene function clusters (S6 Table). Cluster enrichment was performed for the whole list of genes with mutations and the top 10 enriched clusters were picked for protein functional network analysis based on p-value. Multiple testing corrections like Benjamini method was routinely used to reduce false positive functional terms, however, at the same time, its strict cutoff may have disrupted the selection of significant clusters among multiple gene sets[54]. Therefore, although significant pathways were not found from Benjamini correction, gene functions and pathways were selected with p-value analysis (p-value < 0.05, cluster enrichment score > 1.3, refer to Table 1 legend). The purpose of this study was to depict how genetic variants are related to each other in a biological system and to find their associations among enriched functions that may affect the pathogenesis of BRONJ as a group of genes.

**Table 1:** Top 10 clusters ranked by enrichment score and representative terms in each cluster from GSEA results (based on DAVID).

**Nebulin**	**Enrichment Score: 2.84**				
Category	Term	Count	%	P-value	Benjamini
INTERPRO	Nebulin 35 residue motif	3	1.55	6.72E-04	0.09
INTERPRO	Nebulin	3	1.55	6.72E-04	0.09
SMART	NEBU	3	1.55	8.28E-04	0.09
**Basal plasma membrane**	**Enrichment Score: 2.53**				
Category	Term	Count	%	P-value	Benjamini
GOTERM_CC_FAT	basolateral plasma membrane	9	4.64	2.01E-03	0.41
GOTERM_CC_FAT	basal plasma membrane	4	2.06	2.95E-03	0.18
GOTERM_CC_FAT	basal part of cell	4	2.06	4.46E-03	0.18
**Cytoskeleton**	**Enrichment Score: 1.83**				
Category	Term	Count	%	P-value	Benjamini
GOTERM_CC_FAT	cytoskeleton	28	14.43	2.68E-03	0.21
GOTERM_CC_FAT	cytoskeletal part	21	10.82	4.59E-03	0.16
GOTERM_CC_FAT	microtubule cytoskeleton	13	6.70	2.02E-02	0.32
**EGF**	**Enrichment Score: 1.81**				
Category	Term	Count	%	P-value	Benjamini
INTERPRO	EGF-like region, conserved site	12	6.19	3.20E-04	0.13
INTERPRO	EGF-like, type 3	8	4.12	4.89E-03	0.23
INTERPRO	EGF calcium-binding	5	2.58	7.37E-03	0.27
**Ubl & Isopeptide bond**	**Enrichment Score: 1.81**				
Category	Term	Count	%	P-value	Benjamini
SP_PIR_KEYWORDS	ubl conjugation	14	7.22	6.01E-03	0.23
SP_PIR_KEYWORDS	isopeptide bond	8	4.12	4.05E-02	0.42
**Immunoglobulin**	**Enrichment Score: 1.74**				
Category	Term	Count	%	P-value	Benjamini
INTERPRO	Fibronectin, type III-like fold	9	4.64	8.23E-04	0.09
INTERPRO	Fibronectin, type III	9	4.64	1.01E-03	0.08
SMART	FN3	9	4.64	1.88E-03	0.11
**Cell adhesion**	**Enrichment Score: 1.70**				
Category	Term	Count	%	P-value	Benjamini
GOTERM_CC_FAT	proteinaceous extracellular matrix	11	5.67	3.19E-03	0.16
GOTERM_CC_FAT	extracellular matrix	11	5.67	5.40E-03	0.16
SP_PIR_KEYWORDS	cell binding	3	1.55	9.46E-03	0.25
**SH3**	**Enrichment Score: 1.68**				
Category	Term	Count	%	P-value	Benjamini
SP_PIR_KEYWORDS	sh3 domain	8	4.12	5.09E-03	0.23
INTERPRO	Src homology-3 domain	7	3.61	2.70E-02	0.47
INTERPRO	Variant SH3	4	2.06	3.23E-02	0.51
**Protease**	**Enrichment Score: 1.65**				
Category	Term	Count	%	P-value	Benjamini
PIR_SUPERFAMILY	serpin	4	2.06	6.23E-03	0.43
INTERPRO	Protease inhibitor I4, serpin	4	2.06	8.87E-03	0.30
SMART	SERPIN	4	2.06	1.18E-02	0.25
**Motor protein**	**Enrichment Score: 1.63**				
Category	Term	Count	%	P-value	Benjamini
INTERPRO	Dynein heavy chain, N-terminal region 2	4	2.06	6.06E-04	0.12
INTERPRO	Dynein heavy chain	4	2.06	6.06E-04	0.12
GOTERM_CC_FAT	dynein complex	4	2.06	6.37E-03	0.17

We provide a list of all annotation terms in each cluster as S4 Table. Enrichment score is the geometric mean of all the enrichment P-values for each annotations term associated with the genes in genetic variant list from BRONJ patients. P-value of each term in each cluster means the significance of the term enrichment with a modified Fisher’s exact test. Enrichment score of 1.3 is equivalent that average of P-values of the terms in cluster is 0.05. Count and percentage (%) in the table show the number of genes that are involved in the annotation term and the ratio of genes related with the term to total genetic variants. Benjamini is one of the multiple testing correction techniques.

**Fig 2:**
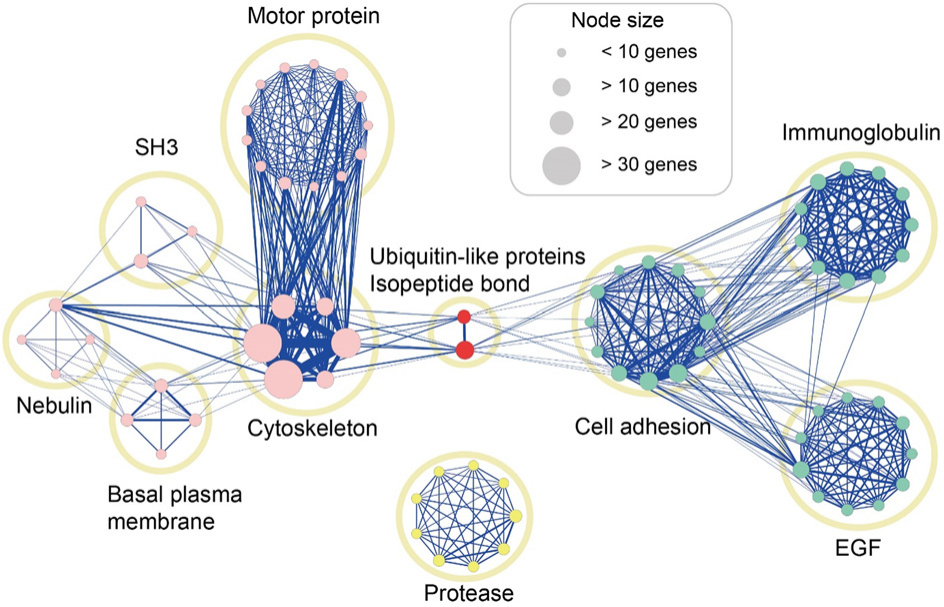
Analysis of gene function enrichment and construction of functional network. Each cluster is represented as a yellow circle, in which nodes show all terms included in the cluster. A representative term was selected to describe each cluster. Node size shows the number of genes mapped in the network. Nodes were color-coded according to their characteristics: genes related to cell morphology (pink), cell adhesion and binding (green), Ubiquitin-like proteins and isopeptide bond (red), and proteases (yellow). If different terms share two or more genes, a link was given. Links express the number of genes shared among distinct terms.

Representative terms in each cluster were listed in Table 1. Protein functional network analysis (Fig. 2) showed numerous mutations in genes affecting cell morphology and cell adhesion and binding. Functional terms and the number of shared genes between two terms in the protein functional network analysis were described using circles, nodes, and lines. Nodes describe functional terms in each cluster, lines express the number of genes shared by each term, node size indicates the number of genetic variants, and link thickness signifies the number of shared genes. Yellow circles represent enriched clusters of gene functions involved in the regulation of cytoskeleton, cell adhesion, and regulation of post-translational modification. These clusters are linked through genes related to post-translational modification such as those affecting ubiquitin-like proteins (UBLs) and isopeptide bonds. UBLs are known to influence substrate affinity, localization, and stability of other proteins by forming isopeptide bonds [55]. Cytoskeletal proteins such as tubulin, actin, and myosin also form isopeptide bonds with the extracellular matrix-associated proteins including collagen, fibronectin, and laminin [56,57].

## Discussion

As it was indicated earlier, the aim of this study was to identify all the biomarkers associated with BRONJ by comparing the genetic information of experimental group with the control group. Generally, in other studies with Whole Exome Sequencing, multiple methods were used to validate the result of variant calling in an effort to minimize the error in the process. However, in this study the validation of variant calling was not performed, because the main focus of this study was to identify new variants associated with BRONJ rather than to identify the location of affected transcript in the mutation. One of the limitations of this study was that although Whole Exome Sequencing is effective in finding the variants responsible for BRONJ, it cannot find the structural information about variants [59]. If one wants to find the affected transcript in variants as is in many researches on cancer or tumor, transcriptome sequencing with different genome sample is necessary. Because this study used saliva samples as a method of collecting DNA, transcriptome sequencing could not be done because it requires tissue samples. The method of whole exome sequencing was adequate for the purpose of this research, which was to detect all the variants associated with BRONJ. However, in in order to study these variants in depth, future study is warranted with transcriptome sequencing, which will allow detection of affected transcript within these variants.

The subjects in the experimental and control groups differed considerably in gender selection. The experimental group had more female participants whereas the control group had more male subjects. Gender bias in the experimental group was accidental and inevitable due to the nature of this study. Currently, bisphosphonates are widely prescribed for patients with osteoporosis. Since osteoporosis is more prevalent in female, bisphosphonate is used more among females. Due to higher use of bisphosphonates in female, higher representation of female patients with bisphosphonate-associated BRONJ was expected in experimental group. Because the incidence of BRONJ is low, the gender difference in incidence remains unknown and needs further study. The effect of gender bias in our experimental group in this study is not clear [50,58,59].

Despite many hypotheses (including remodeling suppression, infection, and angiogenesis disorder), the exact mechanism behind BRONJ is still unclear [60]. Remodeling suppression through inhibition of the mevalonate pathway is the best-known hypothesis. Nitrogen-containing BPs that inhibit the mevalonate pathway in osteoclast interfere with post-translational modification by blocking membrane transport across the endoplasmic reticulum. BPs absorbed by osteoclast act as an inhibitor of farnesyl pyrophosphate synthase, an ubiquitin-like protein, preventing the biosynthesis of small GTPase signaling proteins [2]. These GTP-binding proteins (Ras, Pho, Rac, Rab) are important for cellular growth and are involved in cytoskeletal activity of bone-resorbing osteoclasts [61,62]. Transport across the endoplasmic reticulum is interrupted in the process so that ruffled borders no longer form along the osteoclast cell membrane [26]. As a result, sequestrum is found where necrosis has occurred in the bone remodeling process. As mentioned earlier, BRONJ may be due to combination of predisposing factors and multigentic factors. This study attempted to find a multigenic module responsible for BRONJ [32,35,38]. Of the 201 significant variables found in this study, the highest 10 variants with the lowest p-values were looked at for their effect on BRONJ. Genes ARSD, SLC25A5, CCNYL2, PGYM were among the top 10 variants found. One functionally relevant gene was ARSD, which codes for the protein which participates in bone composition. SLC25A5 is known to code for the protein responsible for transferring energy in the cell and whose mutation may lead to dysfunction in cell metabolism [2]. These genes did not have a direct phenotypical effect on BRONJ but may participate in cell morphology and cell function. It has been reported that transglutaminase regulates the bone remodeling process by forming isopeptide bonds during protein post-translational modification. Transglutaminase is an Ubiqutin-like protein which modulates GTPase activity. The enzyme behaves as a multifunctional protein involved in inflammatory effects through GTP hydrolyzation and protein cross linking. BPs interfere with transglutaminase regulation and inhibit osteoclast activity.

In this study, we found genetic mutations in variants which regulate post-translational maturation. Significant differences between BRONJ patients and the control group appeared in genes related to ubiquitin-like proteins, isopeptide bonds, cell adhesion, and cytoskeleton. These genes are responsible for post-translational maturation and can affect cell differentiation [57]. The results from this study suggest that BRONJ-inducing factors are genetically associated and that BRONJ arises due to the malfunctioning of post-translational modification in osteoclast, leading to the impairment of cell morphology and adhesion.

Despite all statistics regarding BRONJ and BP prescriptions given to osteoporosis patients, the effect of BP use needs to be ascertained by classifying all complications that arise in medical procedures. Among the few BRONJ diagnostic methods are serodiagnosis such as serum CTX (carboxy-terminal collagen crosslinks) and measuring the level of osteocalcin to diagnose the risk of developing BRONJ. Radiologic examination such as bone scintigraphy and MRI are often used in current clinical practice despite their inaccuracy because there is no better diagnostic tool for measuring the risk of developing BRONJ at this point [63,64]. It is thus essential to develop an innovative diagnostic tool for BRONJ.

## Conclusion

BP in the biological system is known to inhibit osteoclastic bone resorption via phagocytosis and internalization by osteoclasts, triggering apoptosis of osteoclasts and eventually inhibiting osteoclast-mediated bone resorption. BPs inhibit the post-translational modification process by blocking the mevalonate pathway in osteoclast and preventing ruffled borders from forming along the osteoclast cell membrane. In this study a significant difference between BRONJ patients and randomized subsamples group was found in genes related to ubiquitin-like proteins, isopeptide bonds, cell adhesion, and cytoskeleton, all of which are involved in post-translational maturation. One may conclude that BRONJ-inducing factors are genetically associated and cause the malfunctioning of post-translational modification in osteoclast leading to the impairment of cell morphology and adhesion. Genetic diagnosis of BRONJ can help clinicians determine appropriate treatment and thus reduce possible complications. Also, post-translational maturation in the mevalonate pathway can be further investigated through genetic research similar to the current study to elucidate the mechanism of BRONJ pathogenesis in detail. Further research with more cases and controls, along with functional animal studies, may produce legitimate biomarkers for early diagnosis of BRONJ.

## Supporting Information

S1 Table List of control variants. (XLSX)

S2 Table List of case variants. (XLSX)

S3 Table Patient information. (DOCX)

S4 Table Summary of the number of reads and coverage. (DOCX)

S5 Table Full list of genes. (DOCX)

S6 Table List of the annotations in each cluster. (XLSX)
