# Supplementary material for: Genetic Investigation of Bisphosphonate-Related Osteonecrosis of Jaw (BRONJ) via Whole Exome Sequencing and Bioinformatics
Source: PLoS One. 2015 Feb 10;10(2):e0118084. doi: 10.1371/journal.pone.0118084 (PMC4337898; doi:10.1371/journal.pone.0118084)
Supplement: S3 Table — (DOCX) [file pone.0118084.s003.docx]

**Table S3.** Patient Information

| Sample ID | Gender | Age | Smoking | Prescribed bisphosphonate | Duration of Prescription |
| --- | --- | --- | --- | --- | --- |
| Exp1 | Female | 71 | NO | Fosamax*, Bonviva** | 7 years |
| Exp2 | Female | 68 | NO | Tybonweekly* | 5 years |
| Exp3 | Female | 79 | NO | Fosaqueen* | 4 years |
| Exp4 | Female | 82 | NO | Fosamax* | 8 months |
| Exp5 | Female | 66 | NO | Ostol*** | 3 years |
| Exp6 | Female | 85 | NO | Fosamax*, Bonviva** | 5 years |
| Exp7 | Female | 71 | NO | Aidbone* | 1 year |
| Exp8 | Female | 78 | NO | Fosamax* | 6 months |
| Exp9 | Female | 72 | NO | Fosamax* | 1 year |
| Exp10 | Female | 73 | NO | Bonviva** | 10 years |
| Exp11 | Female | 90 | NO | Fosamax* | 6 years |
| Exp12 | Female | 55 | NO | Alenmax* | 2 years |
| Exp13 | Female | 67 | NO | Risenex-Plus*** | 7 years |
| Exp14 | Male | 79 | NO | Alendronate | 4 years |
| Exp15 | Female | 71 | NO | Fosaqueen* | 3 years |
| Exp16 | Female | 75 | NO | Fosamax* | 3 years |
| **refer to Alendronate, **Zoledronic Acid, and ***Risedronate* | | | | | |
