# Supplementary material for: Genetic Investigation of Bisphosphonate-Related Osteonecrosis of Jaw (BRONJ) via Whole Exome Sequencing and Bioinformatics
Source: PLoS One. 2015 Feb 10;10(2):e0118084. doi: 10.1371/journal.pone.0118084 (PMC4337898; doi:10.1371/journal.pone.0118084)
Supplement: S4 Table — (DOCX) [file pone.0118084.s004.docx]

**Table S4.** Summary of number of reads and coverage

| Sample ID | Yield (Mbases) | # Reads | % of >= Q30 Bases (PF) | Mean Quality Score (PF) | Mean Coverage Depth | Percentage of reads align on target (%) |
| --- | --- | --- | --- | --- | --- | --- |
| Exp1 | 7,928 | 78,492,298 | 91.67 | 35.77 | 86.44 | 56.16 |
| Exp2 | 5,323 | 52,704,436 | 91.52 | 35.72 | 63.45 | 61.40 |
| Exp3 | 6,661 | 65,954,782 | 91.65 | 35.76 | 78.82 | 60.94 |
| Exp4 | 5,794 | 57,370,070 | 91.45 | 35.69 | 68.92 | 61.26 |
| Exp5 | 6,466 | 64,019,308 | 92.54 | 36.03 | 78.61 | 62.62 |
| Exp6 | 7,150 | 70,789,362 | 92.4 | 35.97 | 82.47 | 59.41 |
| Exp7 | 7,183 | 71,115,752 | 92.37 | 35.98 | 84.59 | 60.66 |
| Exp8 | 6,999 | 69,295,136 | 92.33 | 35.96 | 83.62 | 61.54 |
| Exp9 | 6,879 | 68,105,898 | 92.26 | 35.94 | 82.50 | 61.78 |
| Exp10 | 6,330 | 62,677,148 | 92.41 | 35.97 | 74.44 | 60.57 |
| Exp11 | 6,570 | 65,050,110 | 92.25 | 35.92 | 80.08 | 62.78 |
| Exp12 | 6,317 | 62,549,018 | 92.11 | 35.86 | 77.05 | 62.82 |
| Exp13 | 6,939 | 68,700,752 | 92.19 | 35.91 | 83.41 | 61.91 |
| Exp14 | 8,307 | 82,242,770 | 92.13 | 35.91 | 97.81 | 60.64 |
| Exp15 | 6,736 | 66,694,634 | 92.26 | 35.93 | 80.28 | 61.39 |
| Exp16 | 6,748 | 66,808,832 | 92.31 | 35.95 | 83.21 | 63.52 |
