# Supplementary material for: Genetic Investigation of Bisphosphonate-Related Osteonecrosis of Jaw (BRONJ) via Whole Exome Sequencing and Bioinformatics
Source: PLoS One. 2015 Feb 10;10(2):e0118084. doi: 10.1371/journal.pone.0118084 (PMC4337898; doi:10.1371/journal.pone.0118084)
Supplement: S5 Table — (DOCX) [file pone.0118084.s005.docx]

**Table S5.** Full list of genes

| Gene Symbol | Num Unique Sites | Num Variants | NumTest Samples Affected | % of Test samples affected | % control samples affected |
| --- | --- | --- | --- | --- | --- |
| ABCA8 | 1 | 6 | 6 | 37.5 |  |
| ABCC8 | 1 | 11 | 11 | 68.75 |  |
| ACSM5 | 1 | 10 | 10 | 62.5 |  |
| AGER | 1 | 1 | 1 | 6.25 |  |
| AIPL1 | 1 | 12 | 12 | 75 |  |
| ALDH1B1 | 1 | 12 | 12 | 75 |  |
| ALDH3B2 | 1 | 3 | 3 | 18.75 |  |
| ALDOC | 1 | 2 | 2 | 12.5 |  |
| AMOTL1 | 1 | 4 | 4 | 25 |  |
| ANKS6 | 1 | 4 | 4 | 25 |  |
| ANO4 | 1 | 6 | 6 | 37.5 |  |
| AP000322.54 | 1 | 3 | 3 | 18.75 |  |
| AP000708.1 | 1 | 2 | 2 | 12.5 |  |
| APOB | 1 | 16 | 16 | 100 |  |
| AQP7 | 1 | 12 | 12 | 75 |  |
| ARSD | 5 | 4 | 1 | 6.25 |  |
| ATP1A4 | 1 | 4 | 4 | 25 |  |
| ATP2A3 | 1 | 13 | 13 | 81.25 |  |
| ATP7A | 1 | 9 | 9 | 56.25 |  |
| ATXN7 | 1 | 5 | 5 | 31.25 |  |
| AVPR2 | 1 | 16 | 16 | 100 |  |
| B4GALT5 | 1 | 1 | 1 | 6.25 |  |
| BCL2A1 | 1 | 7 | 7 | 43.75 |  |
| C10orf112 | 2 | 0 | 0 | 0 | 78.57 |
| C11orf34 | 1 | 16 | 16 | 100 |  |
| C17orf80 | 1 | 3 | 3 | 18.75 |  |
| C20orf132 | 1 | 14 | 14 | 87.5 |  |
| C2orf42 | 1 | 4 | 4 | 25 |  |
| CAPN2 | 1 | 2 | 2 | 12.5 |  |
| CCDC6 | 1 | 9 | 9 | 56.25 |  |
| CCDC64B | 1 | 2 | 2 | 12.5 |  |
| CCDC73 | 1 | 2 | 2 | 12.5 |  |
| CCDC83 | 1 | 6 | 6 | 37.5 |  |
| CCL1 | 1 | 3 | 3 | 18.75 |  |
| CCNYL2 | 6 | 76 | 16 | 100 |  |
| CDC27 | 1 | 4 | 4 | 25 |  |
| CEL | 1 | 2 | 2 | 12.5 |  |
| CEP19 | 1 | 15 | 15 | 93.75 |  |
| CFTR | 1 | 3 | 3 | 18.75 |  |
| CHIA | 1 | 14 | 14 | 87.5 |  |
| CHL1 | 1 | 2 | 2 | 12.5 |  |
| CHPT1 | 1 | 4 | 4 | 25 |  |
| CHRNB2 | 1 | 0 | 0 | 0 | 22.22 |
| CLASP2 | 1 | 4 | 4 | 25 |  |
| CNTN5 | 1 | 4 | 4 | 25 |  |
| CNTNAP3 | 1 | 12 | 12 | 75 |  |
| COL3A1 | 1 | 2 | 2 | 12.5 |  |
| COL4A3 | 1 | 9 | 9 | 56.25 |  |
| COX10 | 1 | 0 | 0 | 0 | 50.40 |
| CSMD2 | 1 | 2 | 2 | 12.5 |  |
| CTSH | 1 | 2 | 2 | 12.5 |  |
| CXorf22 | 1 | 10 | 10 | 62.5 |  |
| CYP2A7P1 | 1 | 3 | 3 | 18.75 |  |
| DDB1 | 1 | 2 | 2 | 12.5 |  |
| DDX53 | 1 | 2 | 2 | 12.5 |  |
| DLG3 | 1 | 9 | 9 | 56.25 |  |
| DLGAP5 | 1 | 6 | 6 | 37.5 |  |
| DMD | 1 | 14 | 14 | 87.5 |  |
| DNAH1 | 1 | 3 | 3 | 18.75 |  |
| DNAH8 | 1 | 14 | 14 | 87.5 |  |
| DNHD1 | 1 | 2 | 2 | 12.5 |  |
| DOCK4 | 1 | 12 | 12 | 75 |  |
| DUOX2 | 1 | 2 | 2 | 12.5 |  |
| DYNC2H1 | 1 | 3 | 3 | 18.75 |  |
| ECH1 | 1 | 6 | 6 | 37.5 |  |
| EFCAB3 | 1 | 0 | 0 | 0 | 23.02 |
| EFHC2 | 1 | 4 | 4 | 25 |  |
| EPHA10 | 1 | 7 | 7 | 43.75 |  |
| EPHA6 | 1 | 2 | 2 | 12.5 |  |
| EPHX1 | 1 | 12 | 12 | 75 |  |
| EPPK1 | 1 | 2 | 2 | 12.5 |  |
| FAM72D | 1 | 7 | 7 | 43.75 |  |
| FBRSL1 | 1 | 9 | 9 | 56.25 |  |
| FBXO34 | 1 | 0 | 0 | 0 | 30.95 |
| FMO6P | 1 | 16 | 16 | 100 |  |
| FUT3 | 1 | 12 | 12 | 75 |  |
| GHRL | 1 | 2 | 2 | 12.5 |  |
| GTF3C1 | 1 | 2 | 2 | 12.5 |  |
| GXYLT1 | 1 | 14 | 14 | 87.5 |  |
| HIST1H2BE | 1 | 16 | 16 | 100 |  |
| HLA-A | 1 | 5 | 5 | 31.25 |  |
| HLA-C | 1 | 12 | 12 | 75 |  |
| HMCN2 | 1 | 1 | 1 | 6.25 |  |
| HPSE2 | 1 | 14 | 14 | 87.5 |  |
| HSPA2 | 1 | 2 | 2 | 12.5 |  |
| IL17RA | 1 | 4 | 4 | 25 |  |
| IQGAP2 | 1 | 3 | 3 | 18.75 |  |
| IRF6 | 1 | 0 | 0 | 0 | 26.19 |
| ITPKB | 1 | 2 | 2 | 12.5 |  |
| JMJD7 | 1 | 2 | 2 | 12.5 |  |
| KCNG4 | 1 | 9 | 9 | 56.25 |  |
| KIAA0284 | 1 | 0 | 0 | 0 | 19.05 |
| KIAA1161 | 1 | 15 | 15 | 93.75 |  |
| KLRC1 | 1 | 2 | 2 | 12.5 |  |
| KRT18 | 1 | 5 | 5 | 31.25 |  |
| KRT39 | 1 | 0 | 0 | 0 | 19.84 |
| KRT76 | 1 | 4 | 4 | 25 |  |
| KRTAP10-1 | 1 | 3 | 3 | 18.75 |  |
| KRTAP5-2 | 1 | 6 | 6 | 37.5 |  |
| LAMA2 | 1 | 16 | 16 | 100 |  |
| LAMA4 | 1 | 4 | 4 | 25 |  |
| LIMS1 | 1 | 0 | 0 | 0 | 19.05 |
| LPHN2 | 1 | 3 | 3 | 18.75 |  |
| LRP1 | 1 | 0 | 0 | 0 | 19.05 |
| LRP1B | 1 | 14 | 14 | 87.5 |  |
| MACF1 | 1 | 6 | 6 | 37.5 |  |
| MAGEE2 | 1 | 16 | 16 | 100 |  |
| MBL2 | 1 | 11 | 11 | 68.75 |  |
| MEGF6 | 1 | 6 | 6 | 37.5 |  |
| MIA3 | 1 | 14 | 14 | 87.5 |  |
| MLL3 | 3 | 12 | 9 | 56.25 |  |
| MLLT4 | 1 | 2 | 2 | 12.5 |  |
| MOB3C | 1 | 16 | 16 | 100 |  |
| MPP3 | 1 | 2 | 2 | 12.5 |  |
| MRPL22 | 1 | 5 | 5 | 31.25 |  |
| MRPL23 | 1 | 2 | 2 | 12.5 |  |
| MRPL37 | 1 | 3 | 3 | 18.75 |  |
| MUC16 | 1 | 9 | 9 | 56.25 |  |
| MYH4 | 1 | 13 | 13 | 81.25 |  |
| MYO18A | 1 | 0 | 0 | 0 | 25.40 |
| MYOM3 | 1 | 9 | 9 | 56.25 |  |
| N4BP2 | 1 | 7 | 7 | 43.75 |  |
| NAV2 | 1 | 15 | 15 | 93.75 |  |
| NBAS | 1 | 8 | 8 | 50 |  |
| NEB | 2 | 0 | 0 | 0 | 20.63 |
| NEBL | 1 | 7 | 7 | 43.75 |  |
| NFIC | 1 | 0 | 0 | 0 | 19.05 |
| NOC2L | 1 | 5 | 5 | 31.25 |  |
| NOTCH4 | 1 | 2 | 2 | 12.5 |  |
| NQO1 | 1 | 7 | 7 | 43.75 |  |
| NRAP | 1 | 10 | 10 | 62.5 |  |
| NXF3 | 1 | 2 | 2 | 12.5 |  |
| OBSL1 | 1 | 16 | 16 | 100 |  |
| OR11H1 | 1 | 2 | 2 | 12.5 |  |
| OR1I1 | 1 | 6 | 6 | 37.5 |  |
| OR2T3 | 1 | 15 | 15 | 93.75 |  |
| OTOG | 1 | 3 | 3 | 18.75 |  |
| PABPC1 | 2 | 6 | 3 | 18.75 |  |
| PAX8 | 1 | 2 | 2 | 12.5 |  |
| PER2 | 1 | 2 | 2 | 12.5 |  |
| PKD1L2 | 1 | 16 | 16 | 100 |  |
| PLXNA2 | 1 | 0 | 0 | 0 | 23.02 |
| PM20D1 | 1 | 6 | 6 | 37.5 |  |
| PRTFDC1 | 1 | 2 | 2 | 12.5 |  |
| PRUNE2 | 1 | 14 | 14 | 87.5 |  |
| PYGM | 2 | 8 | 6 | 37.5 |  |
| QARS | 1 | 2 | 2 | 12.5 |  |
| RAD17 | 1 | 0 | 0 | 0 | 18.25 |
| REPIN1 | 1 | 14 | 14 | 87.5 |  |
| RETSAT | 2 | 17 | 9 | 56.25 |  |
| RGS11 | 1 | 14 | 14 | 87.5 |  |
| RTDR1 | 1 | 3 | 3 | 18.75 |  |
| SACS | 1 | 0 | 0 | 0 | 23.02 |
| SCFD2 | 1 | 10 | 10 | 62.5 |  |
| SEC31B | 1 | 9 | 9 | 56.25 |  |
| SEMA4D | 1 | 5 | 5 | 31.25 |  |
| SEMA5B | 1 | 2 | 2 | 12.5 |  |
| SERPINB13 | 1 | 3 | 3 | 18.75 |  |
| SERPINB7 | 1 | 2 | 2 | 12.5 |  |
| SERPINB8 | 1 | 10 | 10 | 62.5 |  |
| SERPINF1 | 1 | 2 | 2 | 12.5 |  |
| SHROOM4 | 1 | 2 | 2 | 12.5 |  |
| SLC24A1 | 1 | 12 | 12 | 75 |  |
| SLC25A32 | 1 | 3 | 3 | 18.75 |  |
| SLC25A5 | 5 | 22 | 11 | 68.75 |  |
| SLC28A2 | 1 | 2 | 2 | 12.5 |  |
| SLC45A3 | 1 | 1 | 1 | 6.25 |  |
| SLCO1B1 | 1 | 1 | 1 | 6.25 |  |
| SOGA2 | 1 | 3 | 3 | 18.75 |  |
| SPEG | 1 | 0 | 0 | 0 | 26.19 |
| SRMS | 1 | 2 | 2 | 12.5 |  |
| SYNPO2L | 1 | 2 | 2 | 12.5 |  |
| TBX4 | 1 | 2 | 2 | 12.5 |  |
| TCF7L2 | 1 | 2 | 2 | 12.5 |  |
| TEKT2 | 1 | 8 | 8 | 50 |  |
| TEKT4 | 1 | 3 | 3 | 18.75 |  |
| TEX26 | 1 | 5 | 5 | 31.25 |  |
| TMC6 | 1 | 12 | 12 | 75 |  |
| TNC | 1 | 13 | 13 | 81.25 |  |
| TOR1AIP1 | 1 | 0 | 0 | 0 | 18.25 |
| TRMT1L | 1 | 0 | 0 | 0 | 19.84 |
| TTC22 | 1 | 1 | 1 | 6.25 |  |
| TTN | 1 | 2 | 2 | 12.5 |  |
| UHRF1BP1L | 1 | 2 | 2 | 12.5 |  |
| UNC93B1 | 1 | 0 | 0 | 0 | 23.81 |
| USP10 | 1 | 2 | 2 | 12.5 |  |
| UTS2R | 1 | 2 | 2 | 12.5 |  |
| VEGFB | 1 | 0 | 0 | 0 | 23.02 |
| VIT | 1 | 11 | 11 | 68.75 |  |
| VNN3 | 1 | 4 | 4 | 25 |  |
| VPS13C | 1 | 3 | 3 | 18.75 |  |
| VPS13D | 1 | 2 | 2 | 12.5 |  |
| VPS41 | 1 | 0 | 0 | 0 | 19.05 |
| VWF | 1 | 3 | 3 | 18.75 |  |
| WDR49 | 1 | 6 | 6 | 37.5 |  |
| WDR67 | 1 | 12 | 12 | 75 |  |
| WDR81 | 1 | 2 | 2 | 12.5 |  |
| WFDC1 | 1 | 0 | 0 | 0 | 22.22 |
| ZCCHC14 | 1 | 0 | 0 | 0 | 31.75 |
| ZNF395 | 1 | 0 | 0 | 0 | 20.63 |
| ZNF782 | 1 | 5 | 5 | 31.25 |  |
